# Emotional straying: Flux and management of women’s emotions in social media

**DOI:** 10.1371/journal.pone.0295835

**Published:** 2023-12-13

**Authors:** Pengpeng Li, Qianru Zhuo

**Affiliations:** 1 Department of Shiliangcai Journalism and Communication School, Zhejiang Sci-Tech University; Hangzhou, Zhejiang, China; 2 Department of College of Communication, National Chengchi University, Taipei, Taiwan, China; University of Rome La Sapienza: Universita degli Studi di Roma La Sapienza, ITALY

## Abstract

In recent years, social media, which has emerged with the core focus on interaction within "acquaintance networks," has gradually been infiltrated by "strangers," leading to the disorientation of many users, especially women, amidst the diverse and intricate social platforms and emotional landscapes. Grounded in the experiential perspective of social media users, this study explores the correlations among woman emotions, satisfaction, and behavior, starting from the standpoint of the impact of social media. Through in-depth interviews with woman cohorts in China, various dimensions such as emotional fluctuations, satisfaction levels, and behaviors in social media were examined. The findings reveal that emotional expression serves as a primary motivation and purpose for users to sustain their engagement with social media. Additionally, emotional masking represents a proactive operational behavior induced by the needs for social relationship maintenance and the accumulation of social capital. Furthermore, emotional management manifests as user-initiated abandonment or shift of social media activities in response to perceived emotional stress. On this basis, a conceptual model integrating woman emotions, satisfaction, and behavior in the context of social media was constructed. The outcomes of this research hold significant theoretical and practical implications for future studies on woman emotions and behaviors, as well as for the development of social media functionalities, content management, public media usage, and psychological health interventions.

## Introduction

Since 2003, the establishment and development of social media have progressed rapidly, with an ever-increasing array of functionalities. As of early 2021, the global user count for online social media communities has surpassed 4.2 billion [1]. Among them, platforms like WeChat, Weibo, TikTok, and Xiaohongshu hold the distinction of having the highest user volume and engagement levels within China [2].

### The intimate interconnection between social media and user experience

The rise of social media has intensified the speed of online information dissemination, while also expanding the possibilities for various forms of interpersonal interaction and channels [3]. Its primary advantage lies in creating a free and relaxed environment for users to express themselves and share. Through instant access, observations, and commenting on others’ updates, thoughts, emotions, and life experiences, users can gain social support, personal identity, and entertainment experiences [4, 5]. As social media usability has increased, users have taken on the dual role of both information producers and consumers. With the improvement of social platform functionalities and the recognition and utilization of platform advantages, an increasing number of commercial advertisements and the rapid emergence of e-commerce have significantly enhanced the influence of social media on individual and societal development [6].

Due to its strong role as a communication link between organizations and potential consumers [7, 8], "user experience" has again garnered attention from relevant interest groups. Among these, social media developers and operators seek to enhance user experience to increase user satisfaction and loyalty, thereby attracting more investment. Governments, businesses, and organizations also select social media platforms with large user bases and high satisfaction levels to achieve information dissemination, establish communication and trust, increase sales, and develop potential consumers [9–12]. Within this trend, the publication and management of social media content, as well as user perceptions and behavioral studies, have become crucial factors influencing the objectives and effects of information dissemination for relevant stakeholders. If social media functionalities or their content fail to genuinely meet user needs or compromise their satisfaction with the platform, it may lead to negative perceptions, evaluations, and reduced willingness to continue using the platform or engaging with content publishers [13].

### The impact of social media on interpersonal communication

An important trend in the development of social media is the gradual dissolution of the interaction ecosystem that was originally centered around "acquaintance" networks. The format of social-shared information has shifted from daily life sharing and personal mood expression aimed at "communicating with acquaintances" to purposes like work promotion, business marketing, and self-promotion driven by "achieving commercial/personal benefits." This shift is altering users’ initial motivations, attitudes, and behaviors towards social media.

It has been noted that personal emotional expression and "self-disclosure" of fragmented life moments on social media can help garner attention from others, release personal stress, and regulate negative emotions [14]. However, if such self-disclosure does not receive the positive responses of praise and encouragement that the discloser initially anticipated, it might lead to negative psychological effects, such as anxiety and disappointment [15]. In the pursuit of more attention and approval, users may unconsciously engage in self-presentation, deliberately shaping a self that differs from their real-life circumstances or is entirely different. Moreover, in recent years, numerous new types of social platforms have emerged, such as TikTok, Kwai, Weishi, and Soul, leading people into an information storm where endless "fresh updates" and "advertisements" inundate their daily lives. Research indicates that information/social overload and factors like information quality and relevance can have a negative impact on individual emotions [16]. Negative emotions during the use of social media can indirectly lower users’ willingness to continue using the platform [17–19].

Further analysis suggests that while using social media might lead to phenomena like social media fatigue, anxiety, depression, or even the fear of missing out, these negative mental states can become drivers that compel users to keep using the platform [15]. This, in turn, forms a vicious cycle on both psychological and media usage levels, exacerbating individual negative emotions and stress, thereby reducing satisfaction with the use of social media. Consequently, examining the development of social media functionalities and content management from the perspective of users’ psychological perceptions and the diversity and complexity of their usage behaviors, as well as interventions for public mental health, holds significant importance for effective media operation and promoting positive societal development.

### Gender disparities in social media usage behavior

Research has shown that women tend to use social media more frequently than men [20, 21]. Regardless of whether in Eastern or Western societies, women are generally considered to be a more "emotional" demographic group [22]. Based on an investigation of social media usage behavior among college students from five universities in Beijing, Lanlan Long found that women often use social media for motives such as stress relief, cognitive attention, and staying informed about others’ updates, while men tend to be more driven by habits and interests, such as online gaming [23]. However, precisely because women use social media more frequently and seek more attention and validation from others, their emotions are more susceptible to the influence of social media content. This susceptibility can lead to negative psychological effects, including feelings of loneliness, disappointment, or even depression, and can potentially jeopardize personal health and well-being [4, 20]. Therefore, by studying the social media usage behavior of women and identifying factors that lead to changes in their psychological and behavioral habits, it may be possible to offer new insights and approaches for public emotion management, psychological health interventions, the development and improvement of social media functionalities, and effective content management on platforms.

### Research questions

RQ1: What are the motivations and patterns behind users’ social media usage?
RQ2: What factors influence users’ satisfaction and behavior in using social media?
RQ3: How are emotions and social media usage behavior interconnected?

Based on the research questions, this study initially reviews and discusses relevant literature on the Uses and Gratifications Theory, social media usage satisfaction, the relationship between emotions and social media usage. Subsequently, the Research Design section outlines the subjects and methods employed in this study. The fourth section analyzes the research findings. In the fifth section, the results are discussed, and the theoretical and practical significance of the study is elaborated upon. The sixth section summarizes the limitations of the research and suggests potential directions for future related studies.

## Literature review

The literature review is divided into two parts: (1) Social Media Uses and Gratifications, providing a brief overview of the Uses and Gratifications Theory, factors influencing social media user behavior are discussed, with particular emphasis on gender differences in social media usage motivations, satisfaction, and behaviors; and (2) Emotion and Media Usage. Focusing on the impact of user emotions on social media usage, changes in emotions during the usage process, and the behavioral changes triggered by emotions.

### Social media uses and gratifications

The Uses and Gratifications Theory acknowledges the subjective needs of the audience as triggers for media engagement. Initial research primarily explored mass media uses and motivational gratifications from the perspective of communicators [24]. Subsequently, attention shifted to the intricate process of "social factors + psychological factors → media expectations → media engagement → gratification of needs" in audience media engagement behavior [25]. With the widespread adoption of the Internet and rapid development of social media, scholars have gradually taken notice of and discussed the contemporary applicability of this theory. They have proposed emotionally oriented audience media usage inducing variables, such as "anxiety alleviation, emotional communication, self-realization, and psychological dominance" [26], as well as diverse dimensions affecting audience media satisfaction, including "user needs," "individual user factors," and "social factors."

Research has categorized user engagement with social media and their satisfaction into various aspects: maintaining connections with others/social collectives, establishing common ground, sharing content, conducting social surveys, browsing social networks and updates from others. The fulfillment of different needs triggers distinct usage patterns among users. If social connections or identification needs are met, it further enhances users’ frequency of engagement with social media and their willingness to self-disclose. Satisfaction with informational content stimulates users to spend more time browsing and seeking out related information [27]. Some studies consider this fulfillment of individual needs as an indicator of measuring satisfaction in social media usage. Perceived user satisfaction and usage satisfaction play a crucial role in the development and management of any new technology, media platform, or feature [28]. The Theory of Planned Behavior (TPB), Theory of Reasoned Action (TRA), and the ABC Model of Attitude have also confirmed that attitudes (including satisfaction) directly or indirectly influence behavior, even determining it. Therefore, by examining the factors influencing individuals’ attitudes towards social media usage (especially satisfaction) and their impact on users’ social media behavior, valuable user experience data can be provided for the development and management of social media functionalities. This contributes to the effective development and management of social media platform features, as well as the positive development of society as a whole.

### The influence of gender on the perception and usage patterns of social media content

Gender studies-related literature confirms that there are notable differences in the complexity of brain structures [29] and cognitive styles [30] between men and women. These differences extend to communication styles, content, and language use [31–33]. Especially in terms of language use and perception, women’s daily communication and information gathering tend to focus on vocabulary related to psychological and social processes, while men lean towards mentioning object attributes and non-personal topics [32]. Women also exhibit a greater emphasis on emotional language [32, 34–36]. This is particularly evident in the expression of positive emotions and the maintenance of emotional connections and social relationships [37–39], whereas men tend to express more "anger" [34, 38].

In terms of cognitive styles, women tend to utilize experiential thinking more, while men lean towards rational thinking patterns [39, 40]. As a result, users of different genders have distinct motivations for using social media and engage in different operational behaviors [41]. Krasnova et al. pointed out that men’s use of social media is often task-oriented, with the purpose of "acquiring information," while women are more inclined to use social media for social interaction and maintaining interpersonal connections [42, 43]. Consequently, in terms of frequency and format of content sharing, the woman population is more active on social media [4, 20, 44], often focusing on themes related to family, friends, and social life, using emotionally rich language, and displaying more self-disclosure compared to men [45]. This implies that the online behavior of woman users is often related to their psychological needs, such as sharing information, receiving responses, recognition, or praise. Burke et al. and Skopp found through survey research on Facebook usage behavior that active interaction with friends on social media increases social support and happiness [46, 47]. Interactive behaviors in social media can fulfill the human need for "social living," benefiting stress relief and overall mental and physical well-being [48]. Given that the use of social media aligns with the emotional and social relationship maintenance needs of women, it further deepens the dependence of more woman groups on social media [49, 50].

### Emotions and media usage

#### Emotions and self-disclosure

People often choose to use specific media and consume media content to express and regulate their emotional states [51, 52]. As a result, the use of social media largely stems from individuals’ psychological need to share or vent personal positive or negative emotions. The act of disclosing personal emotions, thoughts, and experiences to others as self-disclosure [53], and the functions of self-disclosure primarily include self-expression, self-clarification, relationship development, social validation, and social control [54]. Furthermore, the use of online media satisfies human needs for social interaction, including maintaining connections, establishing and developing friendships, pursuing love and a sense of social belonging. Particularly in the context of modern society, where interpersonal relationships are increasingly distant, individuals engage in interactions through online social media platforms to establish a sense of community, share warmth, and experience feelings of love. This helps individuals feel intimacy within social relationships and care from others, thus achieving the goal of soothing emotions and relieving stress [55]. Therefore, if self-disclosure is seen as an effective way for individuals to manage emotions and stress, what factors enhance or hinder users’ willingness to engage in self-disclosure on social media?

According to the perspectives of social exchange theory and social penetration theory, individuals are more likely to engage in self-disclosure on social media if they can gain community trust, emotional support, and social reciprocity. Conversely, the willingness to self-disclose decreases with increased privacy risks. Moreover, According to Edward T. Hall’s High and Low Cultural Context concept, France is more collective (high context) than England. That is also supported by Hofstede which shows the UK having a higher degree of individualism [56]. In other words, different social systems and cultural contexts lead to varying factors that affect individuals’ self-disclosure on social media. So, within the user base of social media in China, what are the factors influencing their willingness to engage in self-disclosure? Do these factors reflect the uniqueness of Chinese culture and social interactions?

Research has indicated that audiences are motivated by seeking pleasurable experiences and tend to choose media and content that enhance or prolong their positive emotions, while reducing or redirecting their negative emotions [57]. Social media, in particular, possesses characteristics conducive to emotional regulation. However, the potential negative emotions it can trigger, such as fatigue, depression, and anxiety, have also gained increasing attention and validation through numerous studies [58–61]. In summary, when users encounter positive/affirmative posts from "others" on social media, and if they engage in social comparisons and perceive others’ lives as more exciting than their own, they may experience psychological disparity. This can potentially lead to feelings of disappointment and the inclination to escape reality, among other adverse psychological states or behaviors. Similarly, when faced with negative posts, users may also be influenced by the unfavorable emotions of others, leading them to experience emotional downturns [62].

Goffman believed that individuals in social groups desire to construct an accepted image in the eyes of others, and therefore, they make extensive efforts in self-performance [63]. Within social media platforms established through connections with acquaintances (especially WeChat), communication often occurs among acquaintances or individuals with prior social interactions. In other words, the image individuals perform on this platform directly influences how others with a personal connection perceive them. The process of interpersonal interaction is intertwined with "impression management," implying that individuals consciously or unconsciously shape an "idealized self" or the version of themselves that others see [63]. Xu Qianli further categorized four types of performances that exist among users of "WeChat Moments": "idealized performance," which primarily focuses on showcasing one’s self-image; "misconceived performance," centered around constructing social status and knowledge; "mystified performance," centered on content detached from the mundane world; and "remedial performance," where individuals mitigate daily mistakes or awkward moments [64]. Through an online survey of Chinese WeChat users, found that woman users are more likely to engage in "ritualistic" usage driven by the need for "entertainment and leisure" [65]. They deliberately construct either perfect or unfortunate selves to gain attention or care from others. However, while much current research explores the factors leading users to abandon specific social media platforms, little attention has been given to the subsequent changes in emotional management and media usage behavior.

#### Media escapism and transition

In fact, despite the continuous growth in the variety and user base of social media, nearly half of these platforms are experiencing saturation, leading to a gradual decline in user activity regardless of gender [66]. Results from the China Internet Network Information Center (CNNIC) surveys reveal that as of December 2018, the usage rate of WeChat Moments had decreased by 3.9% compared to the previous year [67]. Meanwhile, features introduced by WeChat such as "moments visible for three days," "moments grouping," "tatus settings," and "instant video" reflect the phenomenon of "social media escape." In contrast, Sina Weibo has seen an increasing trend in its user base in recent years, indicating a significant tendency of users to "return to social platforms."

Digging into the reasons, the academic community often analyzes the factors behind people abandoning the use of social media from the perspective of social media fatigue. Scholars view social media fatigue as a phenomenon that individuals experience after using social media. When the rate and volume of social information updates surpass users’ cognitive loads, they tend to choose avoidance or reduce exposure [68, 69]. Domestic scholars have also provided similar insights into the impact of social media fatigue on behavior. Social media fatigue is the feeling of exhaustion, weariness, or boredom caused by long-term use of social media, leading to user withdrawal or reduced engagement frequency [70]. System overload, information overload, and social overload can lead to users’ social media fatigue, subsequently reducing their willingness to continue using the media [71]. If social media users experience negative emotions like monotony and fatigue on specific platforms, they gradually decrease the frequency of content sharing on that platform, eventually leading to complete withdrawal or transition to other social media platforms [72].

In conclusion, whether it is social media escape or transition, both are closely linked to the influence of users’ emotions. Emotions, a highly-discussed concept, play a significant role particularly in the association between emotions, users’ social media usage satisfaction, and their usage behavior. This study aims to focus on the connection between emotions and the satisfaction and behavior of social media users, whether they lean towards a more emotional orientation, particularly among woman demographics [73]. Through exploring users’ emotional perceptions of social media content and their satisfaction levels, the study aims to investigate the usage behavior of this emotionally oriented woman demographic on social media, as well as the correlation between their emotional changes during usage and decisions to abandon, transition, or return to specific social media platforms. The objective is to expand the emotional dimension of the Uses and Gratifications Theory Model and provide a refined framework for understanding the interrelationship between users’ emotions, satisfaction, and behavior in the context of the social media era.

## Research design

### Participants and methodology

This study draws from the Uses and Gratifications Theory framework and the ABC Model of Attitude to conduct a qualitative study on "media usage motivations, emotions, satisfaction, and behavior among woman social media users in China." Grounded theory is employed to delve into the subject, using semi-structured interviews to collect primary data. The collected data is then coded incrementally using Nvivo 2.0 software, extracting key concepts, delineating subordinate categories, and establishing associative relationships. The aim is to develop a more contextually relevant model of user emotions, satisfaction, and behavior in the context of social media, by exploring the dynamic correlations between woman users’ social media usage, emotions, and behaviors. This research attempts to shed light on the interplay between woman users’ social media usage, emotions, and behaviors, offering valuable insights for social media feature development, psychological well-being interventions, research, and promoting positive societal growth.

This study focused on woman social media users in China. Leveraging the researcher’s identity as a woman and her extensive network of woman friends who often discuss social media usage and emotional states in daily interactions provided both the research direction and methodological insights. This support was instrumental in efficiently identifying research participants. To mitigate potential "echo chamber" effects and enhance the accuracy and effectiveness of the study’s analysis and outcomes, the following approach was adopted:

#### Multi-Channel participant recruitment

The study initiated participant recruitment in April 2021. The recruitment process utilized a combination of "theoretical sampling," "snowball sampling," and "online recruitment." A portion of participants were selected from the researcher’s personal contacts, who then recommended and introduced additional participants. Additionally, the researcher disseminated recruitment information on various social media platforms (such as WeChat Moments, Sina Weibo, Xiaohongshu, Tiktok) to widen the scope and diversity of participants.

#### Participant screening

Ethical considerations, including informed consent, voluntariness, and confidentiality, were rigorously adhered to while selecting participants based on criteria such as identity, years of social media usage (over five years), and diversity (encompassing different ages, professions, and locations). Data collection was halted once information saturation was reached (i.e., substantial repetition in the data provided by participants). The final sample consisted of twenty participants.

#### Formal interviews

Formal interviews were conducted between May and June of the same year, with each interview lasting approximately two hours. These interviews were organized based on the aforementioned recruitment methods and thorough participant screening.

#### Stringent interview process control and ethical disclosure

As ethical review requirements and independent review units for studies utilizing in-depth interview methods are not yet mandatory in China, this study proactively informed participants about the researcher’s identity, interview objectives, and privacy protection measures in the pre-interview assessment and prior to formal interviews. Before conducting formal interviews, the researcher obtained written / verbal consent from all participants regarding the use of their basic information, interview, and recording materials. Additionally, personal information of participants was anonymized when using interview data.

The interview outline was developed based on the aforementioned literature and was further refined through in-depth probing based on participants’ narratives. Prior to the formal interviews, the researcher conducted pilot interviews with six participants to assess the appropriateness of the interview outline. The interview outline encompassed the following key aspects: personal background information (gender, age, education level, occupation, residence); social media usage patterns (years of social media usage, average daily usage duration, preferred social media platforms); motivations for using social media, types of content posted, user satisfaction, perception of emotions, intention for continued usage on social media platforms; reasons for decreased frequency of use or transition to another social media platform, and personal emotional changes.

### Coding of interview data

Interview data was transcribed into verbatim transcripts and the coding process commenced. To enhance objectivity in data handling and minimize error rates, this study employed the investigator triangulation approach, involving the collaboration of two researchers to jointly conduct the data coding process [74]. Table 1 provides a detailed presentation of the coding outcomes for the interview data in this study. In the open coding phase, researchers disassembled all original data and analyzed each statement. Similar or related concepts were integrated and insignificant concepts with less than two occurrences were removed. Focusing on the semantic relationships between concepts, subcategories were consolidated, resulting in thirty subcategories. In the axial coding phase, the researchers further analyzed and synthesized the thirty subcategories from the open coding process, consolidating relatively independent subcategories into main categories. This step showcased the interconnections between various parts of the interview data. Finally, selective coding was utilized to extract core themes that were most relevant to the research questions and objectives, while encompassing other related concepts. These core themes formed the central concepts and topics to be elaborated in subsequent research analysis [75].

**Table 1:** Interview data coding results.

Primitive statements (initial concepts)	Secondary category	Main category	Frequency of occurrence	Core category
Felt really good and happy in that moment, thought I looked great. Wanted to capture it, whether anyone saw it or not.	Expression and recording	Self-disclosure	49	Expression of Emotions
I’m really afraid of getting old, but posting pictures with beauty filters often makes me believe that this is how I actually look.	Build up your confidence			
There are too many bothersome things in daily life, and the stress is overwhelming. Playing games allows me to temporarily forget about all that. I can immerse myself in the game and it becomes a source of happiness.	Escape from the reality	Entertain-ment experience		
Before going to bed, scrolling through my phone and watching food videos or funny sketches helps me relax, as this is the only time that feels like it’s just for me.	Entertainment tools			
Feeling idle makes me anxious. I feel like I should be doing something productive, so I choose to engage with useful things. For instance, I might watch something like a movie for learning English. It makes me feel like I’m not wasting time.	Compatible use			
When I’m feeling down, I tend to share some melancholic words and songs. Others reach out to check on me.	Brush the presence sense	Social interaction		
There are certain articles that I believe deserve a broader audience, so I’m more than willing to reshare them.	Access a sense of mission			
My main intention is to have those I follow or who care about me notice my emotions.	Directive output			
I’m quite anxious about potentially missing an important message from a friend.	Miss the fear			
Originally, I never used to post anything on my social media feed. However, I kept getting questions from friends about why they couldn’t see my posts. I explained that I wasn’t posting anything at all, but some people did not believe me, which became quite bothersome. So, to address this, I started posting occasionally.	Passive appearance			
Always seeing others in happy situations makes me want the same, even if it’s not genuine. I’ll pretend for a while to feel content.	Bask in happiness	Image shaping	31	Emotional camouflage
I can’t help but want others to perceive me as more accomplished than them.	Create a sense of superiority			
Even if you dislike someone, you often still need to interact with them.	Establish a link	Maintain social relations		
I usually post things related to work. When I’m feeling down and want to post something, I hesitate because I’m afraid my supervisors and colleagues might ask about it.	Work is required			
If I’m positive and cheerful, maybe others will like me more.	Impression management			
Everyone advocates spreading positive energy.	Sub to social norms			
I’m not really keen on letting others know how down I feel, nor do I want their emotions and lives to influence me.	Emotional control	Self-concealment	43	Emotional management
Work and social interactions have already exhausted me. I just want to be alone and quietly daydream.	Social fatigue			
Ads are everywhere, or people are just showing off their kids. There’s not much worth looking at, and I don’t really have anything worth showcasing either.	Focus on content quality			
After spending a lot of time scrolling through my phone, it feels like everyone else is incredibly accomplished while I haven’t achieved much. There’s nothing to post, and I don’t even want to look anymore.	Peer pressure			
If you tell others that you’re not in a good mood, they tend to think you’re just complaining. So, saying anything doesn’t help. It’s better not to say anything at all.	Emotional expression makes no sense			
I’m afraid people will think I’m too pessimistic.	Overoverestimate others’ attention to self			
I post about indulging in food, drinks, and fun all day long. It can really evoke envy, but aside from these things, I don’t know what else to post. It’s better not to post at all.	Consider the self-influence on others			
Sometimes, when I revisit what I posted after a while, it seems pretty foolish.	Self-comparison			
After editing, if I think others might misunderstand, I end up deleting what I wrote.	Worried about others misinterpreting the content			
Just want to jot down the frustrating things that happened today.	Diary requirements	Social media platform transfer		
They’re all posting on that app, so I downloaded it and found it quite good. I can post whatever I want without attracting too much attention from my friends.	Network of acquaintances driven			
They’re all strangers, so I can say whatever comes to mind.	Emotional catharsis			
People around me are increasingly out of touch with me. I need a new social circle.	Seek recognition			
Listening to books or reading comments on the content I’ve listened to, occasionally responding, makes me feel calm and relaxed.	Emotional reconciliation			

## Results

**Fig 1:**
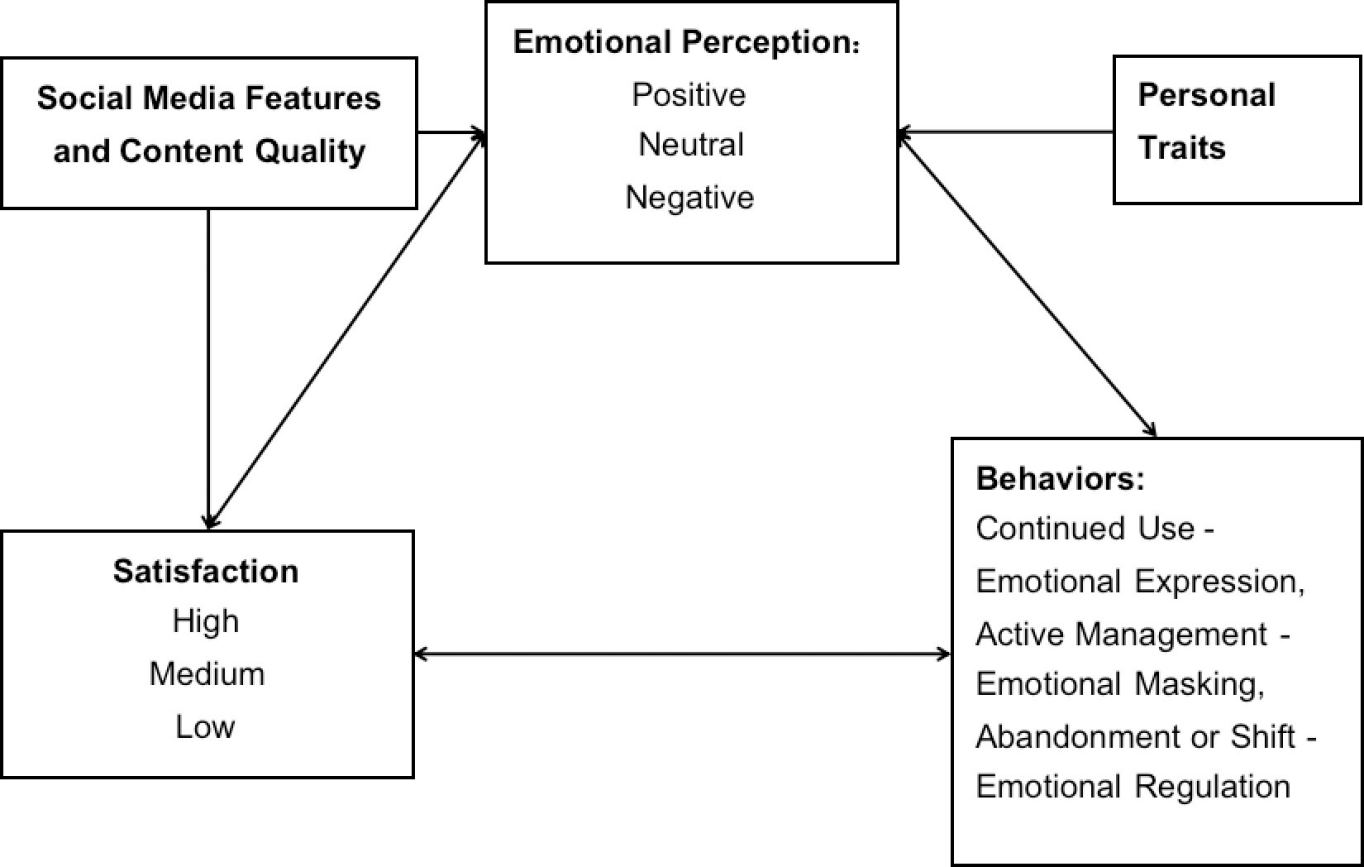
Conceptual model of Women’s Emotion-Satisfaction-Behavior in the context of social media.

The identified core categories in this study are emotional expression, emotional masking, and emotional management. These categories, in conjunction with participants’ social media usage satisfaction and behavioral manifestations, culminate in the conceptual model of user emotion-satisfaction-behavior in the context of social media (Fig 1).

Fig 1 provides a comprehensive depiction of the complex emotional and behavioral dynamics of women in the context of social media. It illustrates that women’s emotional perception of platform content, usage satisfaction, and emotional behaviors are in a dynamically interconnected state, mutually influencing each other. Moreover, emotional perception is not only influenced by users’ individual traits, such as personality and values, but may also be impacted by the functionalities and content quality of social media.

If users themselves belong to the emotionally positive or stable types, they are more likely to experience positive or stable emotions during the process of using social media. Their behavior is influenced, on the one hand, by personal traits or past usage experiences, exhibiting consistent patterns. On the other hand, it is driven by a higher satisfaction with the platform based on positive emotional content perception, rooted in the functionalities and content quality of social media. This, in turn, motivates them to express a willingness to continue using the platform. This continued usage may stem from needs such as self-expression, entertainment, and social interaction, leading them to perceive the platform as a medium for their "emotional expression." Furthermore, the functionalities and content quality of social media also play a role in influencing users’ emotions to a certain extent.

However, if users have a negative personality or are currently in a negative state, they are more likely to experience reverse and negative emotions, leading to reduced satisfaction with their social media usage. Their behavioral patterns may incline towards self-concealment, withdrawal, abandonment, or shifting to other social media platforms. At this point, the functionalities and content quality of social media become crucial in influencing users’ emotional perception and usage satisfaction. On one hand, these factors may intensify users’ negative perception of content and dissatisfaction. On the other hand, they may mitigate or even transform users’ adverse emotions into positive and constructive states. Different emotional perceptions and satisfaction levels have diverse impacts on user behavior, whether it is continued usage, active management, or abandonment and transition to other media platforms.

Additionally, it is worth noting that some users, as their intensity of involvement and immersion in social media usage or content increases, may experience fluctuating emotional states. On one hand, they may express lower evaluations of the social capital accumulation function that social media may bring and exhibit adverse psychological responses triggered by social comparisons with the content. Their individual positive emotions are weakened, leading to decreased satisfaction with media usage. Simultaneously, driven by the potential personal benefits of this function, they actively manage their personal media content, masquerading as an image that aligns with societal expectations or is generally accepted within their social circles. Their social media usage behavior distinctly reflects a significant "emotional masking" appearance. On the other hand, due to factors such as social comparison, social fatigue, emotional contagion, etc., their emotional states may become increasingly negative. This results in a substantial decrease in satisfaction with social media usage, prompting them to engage in self-concealment or transition to other community platforms to stabilize their emotions or effectively manage their emotional well-being.

Building upon the conceptual model in Fig 1 and the results of the current interviews, the following discussion will focus on the core theme of this research: "Flux and management of women’s emotions in social media." The analysis will delve into the emotional behaviors of woman users, specifically emotional expression, emotional masking, and emotional management.

### Emotional expression: The driving force behind continued social media use

#### Intense need for self-expression

From the interview data, it is evident that women’s use of social media is primarily motivated by the need for self-expression, entertainment experiences, and social interactions. Firstly, woman users exhibit a stronger desire for expression compared to men [76]. They often view social media as an outlet for emotional expression, where they can vent negative emotions, extend positive emotions, or document daily moments. The majority of woman participants expressed their desire to capture daily events or emotions in written or visual form, enabling them to revisit and reflect upon them in the future. Looking at the evolution of societal development, humans historically used handwriting to record or disseminate information before technology became so advanced and widespread. This psychological need for recording and expression has merely transformed in form with the advent of the internet and social media. The continuity of this psychological need and behavior suggests that recording and expression aid in releasing personal psychological stress and obtaining positive emotions, such as happiness, contentment, and joy. It also contributes to enhancing personal creativity, communication skills, memory, and stress resistance, ultimately assisting individuals in overcoming challenges and achieving goals [77]. Psychological research reveals that women experience more severe appearance or body-related anxieties compared to men [78]. Worth mentioning is that among the twenty participants in this study, seven mentioned using beauty filters for selfies and posting them on social media, which boost their self-confidence. In other words, this form of self-expression also holds the advantage of enhancing self-confidence. While psychological research suggests that excessive self-presentation could signify a narcissistic personality and often manifests as negative traits like extreme self-centeredness or impulsivity without consideration for others’ feelings [79], being aware of and managing these tendencies can help women avoid being plagued by appearance anxieties in their use of social media and daily life [80]. This interview revealed that these seven participants could exhibit more positive psychological emotions and media usage behaviors, demonstrating fewer instances of negative psychological patterns like social comparison or deliberate self-presentation.

#### Emotional regulation under real-life pressures

Virtual social spaces can cater to users’ entertainment needs, helping them escape the complexities of real-life distractions [81, 82]. Consistent with numerous findings from studies on uses and gratifications, social media serves as a tool for passing time, habitual leisure, temporary detachment from real-life environments, and enjoying satisfying gaming, professional, and communicative experiences [83–85]. Additionally, the fragmented and diverse information on social platforms can fulfill users’ compensatory psychological needs for enhancing various aspects of self-competence. While some research suggests that the constantly changing and updating information in social media might overwhelm users’ cognitive capacities, leading to emotional exhaustion and behaviors of disengagement from such information environments [86], the interview process in this study yielded participants’ remarks indicating that content from sources like WeChat official accounts, WeChat search, and video channels sometimes better aligns with users’ specific/targeted information needs compared to web search engines like Baidu. Through such knowledge supplementation, users can attain a sense of fulfillment and satisfaction, which contributes to a positive and proactive mood as well as behavior. In other words, social media provides more diverse content, and algorithmic features can deliver more tailored content that matches users’ interests and immediate needs, thereby reducing the time spent searching for information, alleviating the pressure associated with information retrieval, and most importantly, granting users the joy of instantly obtaining the information they require [87, 88].

#### The complex psychological dynamics of social interaction

Social media can provide users with a platform for social interaction. This is particularly noteworthy for several reasons. Firstly, the accelerating pace of societal development and the ever-expanding scope of social connections have led to a widespread psychological state known as "lack of presence." In this context, the behavior of seeking a sense of presence through social media, often referred to as "seeking validation," is considered a form of online compensation [89]. The use of social media allows individuals to share and disseminate valuable information with other members of society, engaging in interactions and gaining social support, attention, and a sense of happiness through a single piece of information [90]. Some participants noted that seeing themselves (as individuals or as part of a family) frequently appearing in their "friends’ circle" can garner attention and recognition from others, resulting in positive and joyful emotions. Secondly, more than 80% of the participants indicated that, if their posted content did not receive likes or comments from others, or if it did not meet their expectations, they would experience feelings of disappointment, dejection, and self-doubt. Thirdly, around half of the participants experienced "fear of missing out" psychological pressure. They were afraid of being marginalized or forgotten due to missing updates from others or being unaware of widely known "news" within their social circle. Consequently, they continuously refresh their social media feeds, and this psychological pressure and behavior create a sense of time pressure that they cannot control, leading to feelings of low spirits and discouragement [91]. Fourthly, some participants mentioned that not posting in their friends’ circle for an extended period could prompt certain friends to question them, suspecting that they’ve been blocked or deleted. To alleviate this psychological burden, users might passively post content to clarify the state of their social relationships. Lastly, the use of social media can trigger detrimental psychological effects stemming from social comparison [92]. Throughout the interview process, most participants mentioned experiencing social comparison when browsing others’ (especially relatives’ and friends’) updates. This phenomenon can lead to concerns about appearance, body image, financial status, social standing, self-competence, and even depression or a loss of confidence in life.

In summary, the above analysis indicates that social media users have the right to decide on the content and timing of their information posts, as well as the scope of their network interactions. They can achieve social interaction and psychological satisfaction by constantly updating their own posts and browsing others’ updates. This sense of control over social media and the extension of one’s online connections help alleviate the stress and negative emotions brought about by real-life pressures [93]. Interviewees seek to express their emotions through self-disclosure, entertainment, and social interaction within the online community, as a way of self-care within this virtual space. However, when an individual’s use of social media shifts from being active to passive, they may experience feelings of disappointment, fatigue, self-doubt, and a sense of being lost [94]. Although users with a stronger sense of self-control can use social media correctly and freely, most of the woman participants in this study expressed that the interactions in this virtual space lack the nonverbal cues and warm experiences of in-person communication. It is easy for them to misinterpret others’ opinions about them or the outcomes of interactions, leading to self-doubt and self-negation. This can result in escalating negative emotions and even have a detrimental impact on their daily lives.

### Emotional masking: Proactive management on social media

The habit of dividing one’s living area into "front/backstage" exists across all levels of human society. The front stage is deliberately kept clean and organized to receive guests, while the backstage is relatively more free and casual for personal use [95]. This phenomenon, in Goffman’s perspective, is aptly referred to as the "dramaturgical metaphor." In other words, individuals on the front stage engage in idealized self-presentation to meet or cater to the audience’s expectations of their social role, while on the backstage, they engage in self-examination and analysis, adjusting themselves to return to their authentic state. Applying this view to the self-presentation patterns of woman users on WeChat Moments, it is evident that most interviewees consider the feature. Moments to be a relatively private space for communicating with close friends backstage. However, as the number of "friends" increases, many weak ties occupy this space, causing individuals to contemplate how their posted content aligns with others’ perceptions and their own self-image. The instinct of impression management compels people to shift from the relaxed state of the backstage to the tense and guarded state of the front stage [75]. Individuals carefully present or reshape themselves to leave specific impressions on the audience [96]. The outcome is that social media users are compelled to display the most appealing version of themselves to others, seeking specific evaluations and social capital [97].

From the interview data, it is evident that woman respondents often have motivations related to image cultivation and maintenance of social relationships. They contemplate, modify, and adjust the format of the content they intend to post. Initially, the increasing openness of social platforms provides individuals with more opportunities to learn about the life situations and quality of life of other social members. However, due to this very reason, individuals evaluating themselves might experience constraints from social comparisons. Around sixty percent of the interviewees indicated that they engage in comparisons to some extent while browsing social media. Upon perceiving disparities, they undergo self-reflection and even experience feelings of jealousy. Particularly within WeChat Moments, which mainly consists of acquaintances, members share similar backgrounds, education levels, or income. This implicit peer pressure of parallel comparison, as opposed to upward or downward social comparisons, more easily triggers negative comparisons among users, leading to negative emotions of envy, jealousy, and resentment [75]. Driven by this negative social comparison psychology, users tend to modify their self-image further, aiming to gain an advantage in their friend circles. Alternatively, they might experience self-doubt and self-denial due to the realization that they lack the weapons to surpass others (here referring to personal achievements, relationships, or work performance). Ultimately, this intensifies their negative emotions, possibly even leading to a loss of hope in life. This phenomenon is particularly pronounced within the young woman demographic.

Furthermore, respondents indicated that they consider the quality of their content before posting on social media, often enhancing it through editing images, refining captions, and formatting to elevate its cultural and aesthetic appeal. An interesting phenomenon observed within WeChat moments is that respondents tend to categorize their audience before making content accessible, aiming to reduce unnecessary complications, such as sharing work-related content only with colleagues. However, this process introduces psychological pressures of fatigue and concern about potential misunderstandings, which escalate as the number of friends increases [75]. Additionally, after carefully crafting a self-image on social platforms, individuals need to continuously maintain this image. However, lacking relevant content or topics can trigger anxiety, leading to doubts about self-authenticity and even identity crises. More importantly, some respondents believe that their real-life selves diverge from their online personas, leading to a fear of being discovered by their so-called friends. As a result, they end up embodying their online personas in the real world, trapped in a cycle of social media dependence, and experience social fatigue, exhaustion, and emotional low points. Another noteworthy point is that around twenty percent of the respondents tend to post content related to collective behaviors out of a sense of perceived conformity. For instance, in cases of significant societal events, such as national holidays, the Olympics, disasters, donations and other philanthropic causes, individuals may not be particularly interested in or fond of the topic. However, if their friends are collectively engaging in the discourse, individuals might repost, like, and comment on related information to join the conversation. This participation helps alleviate potential fears of being socially isolated due to not conforming to collective behaviors.

Overall, woman users in social media tend to engage in self-presentation and reshaping of their personas due to their concern for societal (others’) evaluations. However, this self-presentation behavior, which involves masking one’s true self, can lead to a loss of genuine self-awareness, while burdening users with unnecessary psychological stress and social pressure, subsequently impacting their emotions and quality of life. It can be argued that while social media offers individuals a platform for positive self-presentation, the escalating tendency towards habitual self-disguise can gradually erode the courage to face one’s true self and others [98]. From another perspective, humans are species with subjective agency. Besides actively perceiving the objective world, they also possess the capacity to transform the objective world guided by their perception. In other words, individuals, upon recognizing the potential negative effects of social media usage, can adapt their media behavior or seek methods to restore normal levels of emotions, mental states, and cognition. This is what we will analyze in the following concept of "emotion management."

#### Emotion management: Reasons for abandoning or transferring to other social platforms

*Self-concealment*. Presenting genuine life statuses or venting negative emotions can lead to excessive concerns, unnecessary worries, or reproaches from others, such as family, friends, colleagues, or superiors.

It can also result in the accumulation of negative memories for oneself. As mentioned earlier, the dynamic posting on social media often serves as a mere daily record, reflecting an individual’s current psychological state. While receiving attention, care, and greetings from others can provide social support and a sense of presence, it can also impose greater pressure on individuals to explain themselves to the outside world. This ultimately does not contribute to the alleviation of stress and negative emotions. Some respondents indicated that they have an urge to express during emotional fluctuations. However, considering the possible reactions from family members’ concern, friends’ sympathy, or others’ negative perceptions of one’s ability to cope, this immediacy may be suppressed. Furthermore, reflecting on these posts later might reveal one’s immaturity at the time, leading to reduced expression desire and frequent cases of editing and deleting. In addition, the common behavior resulting from this psychological trigger includes periodically deleting or setting previously posted content as visible only to oneself.

Prolonged immersion in social media can be influenced by the media content and the emotions and life statuses of others.

According to the theory of emotional contagion, people’s emotions can be influenced by the emotions of others, resulting in experiencing the same emotions as others [99]. Most respondents mentioned the strong emotional contagion effect within social media during the COVID-19 pandemic. They expressed that during that period, they felt immense panic because everyone was sharing negative information and expressing fear. Similarly, if positive information or "positivity," such as the Olympics or national holidays, was being conveyed by everyone, individuals would feel inspired, hopeful, joyful, and satisfied. Similar findings were also observed in Kramer et al’s research, indicating that when individuals see fewer negative emotional messages posted by their social media contacts, they also post fewer negative emotional messages [100]. This finding suggests that individuals’ perceptions of others’ emotions and collective norms can affect their own emotions and behavior. Consequently, respondents stated that they would choose to conceal or withdraw from such media spaces to gain tranquility, as they did not want to be influenced by others’ or collective emotions and norms. Simultaneously, individuals also tend to self-conceal due to concerns that their posted content might negatively affect others’ psychological and behavioral responses.

The constant influx of updates in social media leads users to experience social fatigue and weariness, subsequently decreasing their engagement and willingness to participate.

The proliferation of weak-tie friends and commercial invasions in platforms like the friend circle on WeChat exacerbates the overload of information and social interactions. Nearly all respondents believe that there is an increasing amount of irrelevant or commercial information in social media (especially WeChat friend circles), which not only intensifies the pressure of selecting useful information, but also leaves them fatigued from the effort of fully browsing through content out of fear of missing something important. Respondents who are in the workplace also noted that the friend circle is becoming a tool for company branding and commercial promotion. As a result, they feel a loss of autonomy over their use of the friend circle, being compelled to passively post content required by their superiors. Moreover, they find themselves obligated to "like" work-related content to demonstrate their commitment to their jobs to colleagues and supervisors. The once emotionally resonant action of "liking" has gradually faded into an implicit form of commercial social currency, leading individuals to grow tired of traversing both online and offline realms in a virtual and detached social landscape.

In connection with the previous analysis, as social platforms become increasingly dominated by commercial activities, their content loses its value for users seeking leisure, entertainment, and stress relief.

Respondents indicate that the excessive presence of commercial advertisements and "showcasing children" content within social networks lacks informational value. They are unable to derive psychological or emotional satisfaction from such content. Moreover, some personal announcements (academic achievements, work accomplishments, personal relationships, etc.) trigger feelings of self-doubt and confusion due to comparison, thereby diminishing their willingness to browse and engage with such content.

*Media shift*. Users initially have a significant degree of autonomy when engaging with a particular social media platform. They can freely express personal emotions or opinions according to their own wishes without being easily constrained by external factors or crafting content to cater to others and societal norms. However, as their personal social networks expand, social spaces become commercialized, and individuals become more aware of the behaviors and norms of others and collectives, their control over social media gradually diminishes. This struggle between reliance on and being controlled by social media leads to a sense of self-disorientation and prompts behaviors of self-concealment, escape, and shifting to protect themselves from intrusion.

Taking a closer look, participants experience something akin to a medical withdrawal reaction in the short-term process of controlling or reducing self-expression and pretense. This reaction involves being increasingly entangled by the fear of missing out and sinking into a sense of melancholy, prompting them to once again drift back into the social media environment. However, if they successfully endure this process, users find that their emotions and daily life gradually return to normal, leading to more positive emotions and a sense of happiness. Some participants mentioned that when they refrain from browsing or posting on their friend circles, they prioritize sharing their needs or emotional challenges with their close friends, family, or "small groups" (meaning groups of friends, usually within five people). Real-life friends and deep conversations or jesting with them provide more comfort and solace than the fleeting attention and consolation from the virtual "liking" interactions. However, a more noteworthy phenomenon is that over seventy percent of respondents indicated that they feel more relaxed and at ease when conversing with strangers in the online space. They also seem to worry less about comments from strangers and find it beneficial to alleviate negative emotions or maintain emotional stability influenced by strangers’ lives or emotional states. In summary, women tend to choose self-concealment due to the perceived adverse effects of habitual social media on their emotions. Yet, they also continually explore, attempt, or develop other means of self-expression and social functions in order to satisfy their impulses and needs for self-expression.

Taking WeChat Moments as an example, while extending users’ social networks, it also weakens the emotional satisfaction that the original "strong ties" in their social relationship network could provide, leaving people connected but still feeling lonely. Most respondents mentioned that they still frequently browse through their friend circles without commenting or liking any posts. Nevertheless, their emotions are still influenced by the content they view, leading to feelings of joy or sadness, contentment or jealousy, relaxation or anxiety. They have an urgent need for a space where they can authentically express themselves and release negative emotions. Some respondents even mentioned that they shift their attention to previously abandoned social media platforms, such as QQ Space, Renren, or Sina Weibo, to document their daily lives, vent emotions, or reconnect with long-lost friends. They may also transition to new media platforms, such as TikTok, Kwai, or Wesee to seek relaxation, enjoyment, and a sense of belonging. Delving into the interview content, the participants’ use of and emotional changes in media other than WeChat Moments can be summarized into the following three patterns:

*Reverting to previously abandoned media environments*. After a significant increase in the popularity of WeChat Moments, the popularity of Tencent QQ, Sina Weibo, and the once-popular Renren Network (formerly known as Xiaonei) among college students sharply declined. However, with users feeling a reduced sense of control and satisfaction in WeChat Moments, some individuals began tentatively returning to previous media environments. Some interviewees mentioned that revisiting old media environments provided them with a warm and pleasant experience related to "usage memories." They expressed feelings of happiness and satisfaction. Within these old environments, users engaged in communication and interaction after seeing updates from many long-lost friends, rebuilding friendships and gaining support and social satisfaction. Additionally, some Weibo users created a "yearly calendar tree hole" on their personal pages, meaning they posted a dynamic (mostly with a year label) and irregularly shared personal feelings. This "recording" behavior is often inconspicuous to both friends and strangers, and it does not influence others, allowing users to achieve psychological satisfaction in expressing their authentic needs and daily recording desires.

*Transitioning to emerging media platforms*. Some respondents, due to their friends’ usage recommendations, exposure to commercial advertisements, or endorsements by celebrities, turn their attention to specific media platforms. Their initial satisfaction with these platforms may lead to a sense of immersion and subsequent negative psychological effects. In general, the most frequently mentioned platforms by respondents include TikTok, Kwai, Xiaohongshu, Instagram, Soul, Meipian and "Oasis. From the perspective of uses and gratifications, these platforms tend to fulfill users’ desires for leisure, escapism, and authentic self-expression. Users often engage with content by participating in discussions and seeking validation through comments, all while minimizing the potential for negative emotions stemming from disagreement or criticism of their viewpoints or stances. However, some respondents also believe that their habit of immersing themselves in social media or browsing strangers’ updates has become a daily routine. While this behavior helps alleviate real-life stress, it brings along a burden of media consumption. In other words, they find it difficult to control their urge to browse, leading to anxiety and self-blame.

*Developing alternative media spaces not primarily focused on social functions*. In this context, many respondents mentioned media platforms, such as Ximalaya, NetEase Cloud Music, and Keep. The main reason for choosing these platforms is that they are driven by users’ personal interests, such as listening to audiobooks, music, or engaging in fitness activities. These platforms were not initially designed with social interaction as their core operation, but their inherent comment functionality has naturally evolved into a space for exchanging opinions. Most respondents believe that connecting with online peers who share similar interests and needs allows for better understanding of their current selves. Through the exchange of opinions in the comments section among diverse members, individuals are more likely to embrace their imperfections and cultivate mental resilience and positive behaviors to overcome challenges in real life.

Overall, the reasons behind individuals reducing their frequency of use and exposure on social media are diverse. Apart from the commonly discussed and studied phenomena of social fatigue and exhaustion, users’ desire for higher quality content on social media, peer pressure, self-comparisons, concerns about others misinterpreting their content, considerations about their potential impact on others, and more, all contribute to users engaging in self-concealment on social media. Additionally, users may choose to transition to familiar network platforms, share personal life updates and emotional release, establish new social circles, and achieve emotional resolution, leading them to use or explore other platforms for their social interactions. However, as previously mentioned, users are subjective individuals with agency, and their behaviors are not static. Whether users feel pressure and evaluation from others, experience social fatigue, exhaustion, engage in social comparisons, or encounter imbalances in social norms within any social media environment, these factors may lead to new instances of self-concealment and a shift to other social media platforms. This is all in an effort to regain autonomy and control over their online experiences.

## Discussion

This study attempts to bridge the gap between the Uses and Gratifications Theory and emotional research literature by situating them within the context of social media. It aims to predict the social media usage behavior of woman users based on their perceptions of platform functionality, content, emotions, and satisfaction. The primary objective is to explore the dynamic correlations between the emotions, satisfaction, and behaviors of woman social media users, thereby extending the traditional theoretical models with an additional dimension of emotions.

The research methodology involved conducting in-depth interviews with twenty Chinese woman social media users to gather their personal experiential data. This data was then coded and analyzed using content analysis methods and tools. The three-level coding of subjective descriptive information from participants was achieved through iterative dialogues with the literature and repeated deliberations among coding personnel. This process aided in the establishment of a conceptual model centered around emotions, satisfaction, and behavior within the context of social media.

This study contributes significantly to the fields of social media functionality development, content management, and user mental health intervention in the following ways: Firstly, it expands the dimensions of existing literature on the Uses and Gratifications Theory. In the context of social media, the traditional theory does not adequately depict user profiles and behavior traits. Thus, this study emphasizes the exploration of diverse factors such as platform content, dynamics, user perception, and satisfaction when discussing user perceptions and behavioral diversity [101]. Secondly, employing in-depth interviews allows for the collection of diverse, complex, and dynamic experiential data from users. This addresses the limitations of big data analysis results in overlooking individual information. Contemporary new media research often leans towards using big data mining methods, which generalize conclusions through data modeling, classification, and analysis. However, in-depth interviews with users provide more authentic and in-depth research data that may have been filtered out by big data analysis, thus enhancing the acquisition of important information [102].

### The impact of social media functionality and content quality on user attitudes and behaviors

Based on the findings of this study, it can be observed that current woman users of social media platforms are often motivated by goals such as self-expression, social interaction, attracting attention and approval, and building social capital. The functionality and content quality of social media directly impact users’ emotional perceptions and satisfaction levels, further influencing their usage behavior. Initially, some users continue to use or rely on specific social media platforms due to the positive emotions and satisfaction derived from the content and interactions within the social community. A significant difference from previous literature is that this study highlights woman users’ focus on their emotional perception and genuine expression needs [103] and to some extent demonstrated the negative impact of social media on the emotions of woman users [104, 105]. However, these studies have not fully explored the changing patterns of usage behavior among woman users who seek to express their emotions on social media and are less concerned about social interactions. This study addresses certain gaps in the field and discovers that woman social media users have a strong desire for emotional expression or mood sharing. This need is a significant driving force behind the rise of social media. However, as more and more complex social relationships are introduced, the satisfaction (or lack thereof) of this need becomes an essential factor influencing users’ media usage behavior.

### Social capital and user media usage patterns

Some users engage in genuine emotional masking and actively curate their social media content due to the perceived social capital generated by the interactions and content on social media. Based on existing literature, on the one hand, the content posted by social media users often tends to moderate highly polarized emotional expressions, which can be categorized as neutral emotions when subjected to machine data mining [106]. On the other hand, social media users tend to hide their true emotions due to various social factors, which is a crucial limitation for most studies that employ data mining methods to extract emotional information from social media users [103]. This study reveals the complex and sometimes contradictory associations between the content presented on social media and users’ psychological states.

### The complex association between woman emotions and social media usage behavior

Some users may experience negative emotions such as social comparison, perceived social norms, information overload, and social fatigue due to the content and interactions on social media. These emotions can lead to feelings of annoyance, abandonment of familiar social media platforms, migration to other platforms, or the development of non-social platforms to achieve positive and constructive experiences for effective emotional management. This finding reflects the concern and need for emotional monitoring and management among Chinese women who use social media. Kim et al., in their research comparing the motives for social media use among college students in the United States and South Korea, found that South Korean students emphasize obtaining social or psychological support from existing social relationships, while American students are relatively more focused on seeking entertainment [107]. From a similar perspective, this study also reveals that participants often entrust their self-perception to online peers and reshape themselves through others’ evaluations. Their emotions are highly susceptible to disturbances from online content, comments, and other interactive behaviors. While some participants are capable of recognizing the edge of emotional turmoil and subsequently abandoning or shifting their media usage, their emotions often remain negative for a period after such abandonment or transition. Among these, those who reduce their media usage frequency or withdraw from a particular social media platform can experience feelings of fear of missing out, emotional volatility, and self-disorientation. On the other hand, users who migrate to alternative social media platforms use self-expression as a means to vent their negative emotions. However, with increased usage frequency, interactions with "friends," and engagement with strangers on the new platform, users can once again experience social norms, comparisons, and fatigue, leading them to a cycle of media use and transition. Typically, users do not directly express their content and emotional perceptions or satisfaction on a specific social media platform. In other words, social media platforms often lack a channel for users to promptly communicate their usage experience, which is also an essential aspect that contemporary smart computing and big data mining methods cannot access. This also underscores an important indicator for the development and construction of social media. Additionally, individual traits also play a significant role in shaping self-emotions and media usage behavior [108]. Based on these research findings, this study also constructs a conceptual model of Women’s Emotion-Satisfaction-Behavior within the context of social media. Furthermore, through this research, it is evident that the construction of social platform features and the utilization and updating of media technologies underscore the dynamic shaping process of technology, individuals, and society. Similarly, changes in users’ social media usage behavior drive the continuous evolution and enhancement of features in social media platforms.

### Theoretical contribution—Exploring women’s emotions and behaviors in the context of social media through in-depth interviews

This study makes theoretical contributions in two main aspects. Firstly, we have developed a conceptual model that focuses on the emotions, satisfaction, and behaviors of woman users within the context of social media. This model extends the existing Uses and Gratifications Theory from the realm of traditional mass media, thus contributing to theory. Secondly, at the methodological level, we have contributed by employing in-depth interviews to collect and analyze user experience data within the realm of social media. This approach helps overcome limitations and shortcomings associated with the more prominently studied data mining methods in this field.

In today’s rapidly evolving society and technology landscape, people increasingly seek psychological and emotional fulfillment within the context of material abundance. While social media, which emerged in response to these changing times, can serve as a compensatory tool for social development and human needs, it also comes with inherent drawbacks of technology-mediated communication. These drawbacks include constantly interconnected but fragile social networks, intensified social comparisons, information overload, emotional contagion, online violence, and more, all ultimately leading to contradictions and a sense of loss in self-perception. Through this research, it has been observed that what was once a virtual space in social media is becoming more intertwined with real-world social networks. Users’ self-perception is increasingly based on social evaluations and expectations, requiring continuous updates and maintenance of their online "self" to achieve unity between the "public self" and the "private self" in real life. As a result, users are gradually losing control over the dominance of media, leading to an inability to find satisfaction in freely expressing themselves, relieving emotions, and managing stress through media usage. This situation can lead to emotional turmoil and even impact daily life and interpersonal interactions.

Improving the construction and management of social media functionalities based on user experience is one effective method in the digital age to meet contemporary individuals’ micro-social needs while avoiding social stickiness. It also assists relevant interest groups in achieving commercial benefits [109]. This paper takes the Uses and Gratifications Theory that originated in the era of mass media and situates it within the context of modern social media. It connects this theory with literature related to emotions, attitudes, and behaviors in public social media use. Through a comprehensive analysis, it is found that there might be correlations between the content, functionalities of social media, and motives, emotional perceptions, satisfaction, and behaviors of users [28, 110].

In this study, we expand our understanding of the dynamics and diversity of emotional satisfaction and usage behaviors of Chinese women users in the context of social media. Existing literature on Chinese social media user behavior indicates that factors such as maintaining social connections, building and nurturing friendships, gaining love and a sense of belonging, as well as managing emotions and relieving stress, contribute to continuous media use [55]. Conversely, overload of social media system functionalities, information, or social interactions, leading to social fatigue, tiredness, boredom, frustration, and exhaustion, are the primary factors that reduce user frequency and willingness to engage with social media [70, 72]. However, these studies did not focus on the dynamic correlation between user emotions and their media usage behaviors. Worth noting is that customer experience during service interactions has been widely discussed [111], yet the detailed linkage between user experience, particularly emotions and behaviors, in the context of social media has received limited exploration. Some studies have leaned towards using data mining methods to analyze gender-based differences in emotional dimensions presented in social media [103], but limitations persist at the methodological level.

Existing literature has identified user experience as a key influencer of behavior. Our study takes a step further to emphasize that in the woman population with higher media usage frequency, platform functionalities, content quality, as well as emotional perceptions have significant impacts on their media experiences, satisfaction, and subsequent usage behaviors. Although research on user emotions, satisfaction, and behavior has been discussed in various fields or from different perspectives, a comprehensive framework for understanding the relationships between these factors has yet to be established. Building upon this, our study not only discusses and supplements existing literature results but also establishes connections and proposes a conceptual model of user emotions, satisfaction, and behavior in the context of social media. Future research could further apply this conceptual model to different cultural backgrounds, diverse identity and gender groups, or various media contexts. Quantitative statistical methods could be employed to validate the model’s fit and stability, providing empirical evidence and theoretical support for continued applications in subsequent research, media management, and service enhancement.

Another major contribution of this study lies in its approach of not merely chasing after novel or trendy research methods, but rather employing a semi-structured in-depth interview methodology. Based on the literature, an interview outline was formulated, and through deep probing based on the interviewees’ responses, a clear understanding of the dynamic relationship between their emotions, satisfaction, and behaviors during social media usage was established. Existing literature, particularly in China, has generally recognized the diverse impact of social media on user emotions. Some studies leaned towards using data mining methods to detect the emotional dimensions of user expression on social media, while others, though employing in-depth interviews, did not further explore the connection between user emotions and subsequent media usage behaviors and changes. This study attempts to establish a dynamic structural relationship between emotions, satisfaction, and behaviors of woman users on social media. The study’s universality and dynamism are demonstrated within the interviewee data, which is an uncommon aspect in existing relevant studies in China. This research approach can also be applied to existing studies as a complementary method to data mining, providing more genuine and comprehensive research data.

### Practical insights

#### Development and maintenance of social media features

This study affirms that women’s use and reliance on social media stem from the positive emotions and experiential satisfaction derived from platform functionalities and content, which facilitate self-expression, entertainment, and social interaction. On the one hand, the act of freely writing within the social space allows users to achieve psychological value through self-disclosure, self-presentation, and self-reflection. On the other hand, social media platforms provide users with the means to develop, establish, and maintain social capital, aiding in expanding social networks and maintaining existing social circles. By sharing personal updates and information content with friends, users can cultivate a sense of social belonging and improve others’ impressions of themselves [100]. When individuals experience feelings of depression, sadness, or insecurity, interacting with friends and acquaintances on social media can provide emotional support, knowledge, information, and practical assistance to alleviate negative emotions [84]. In other words, the use of social media can beneficially assist users in regulating negative emotions, fostering a sense of social belonging and security. Additionally, social media offers users interesting and entertaining content that, compared to traditional media, is more immediate and interactive. It can help users temporarily escape from real-world pressures, relax their mood, pass the time, and attain a sense of happiness and psychological satisfaction through entertainment experiences [84, 112]. In essence, if social media functionalities and content can consistently fulfill users’ psychological, social interaction, and entertainment needs, users will be inclined to increase their self-disclosure and willingness to continue using the platform based on the positive emotions and satisfactory media experiences they gain. Conversely, if these needs are not met, users might engage in self-concealment, withdraw from the platform, or migrate to other social spaces to seek the aforementioned satisfaction.

Of greater concern is the current trend towards increasing diversity in social media platforms and their functionalities. Developers are becoming more attuned to users’ demands for "emotional expression." As social media, driven by commercial interests, evolves to cater to users’ ever-changing needs, algorithms are being integrated to dynamically adjust and deliver relevant information content to users in real time. Additionally, the gradual introduction of commercial advertising is taking place. The outcome of this development may lead users into an "echo chamber" environment, which not only hinders users’ objective understanding of current events but also amplifies collective negative emotions such as anger, depression, and fear, thereby exerting detrimental effects on individuals and society as a whole. Consequently, social media developers need to be acutely aware of the drawbacks associated with algorithms and responsibly rectify media operation mechanisms, in order to foster a positive online environment and socially responsible social platforms.

#### User media usage

This study underscores that women tend to be more emotionally inclined than men. They are more attuned to social norms, the emotions of others, and are prone to social comparison. Their authentic self-expression or presentation might be concealed due to the perception of social norms or transformed into self-presentation due to social comparison. The outcome could be a relief from negative emotions through the acquisition of social capital, a sense of belonging, and entertainment experiences, or an exacerbation of negative emotions due to social fatigue and a stark contrast between virtual and real life. In fact, numerous studies have confirmed that social media users are inclined to present themselves in favorable ways, such as being superior, happy, or perfect. [113, 114]. However, it is important to recognize that social comparison in the online space is often meaningless and distinct from reality [92, 115, 116]. The consequences of comparing oneself to others are typically negative, including depression, low self-esteem, and decreased happiness [115–117]. In his book "Escape the Marginal Life," Hu Zhan Gao suggests that we need to accept our imperfections and reduce comparisons with others to attain a relaxed and content life [118]. Users should have a more rational understanding of their emotional dynamics and the factors that trigger emotional fluctuations in order to objectively evaluate and use social media. By facing and continually reflecting on their relationship with social media, users can use it moderately to regulate their emotions and avoid being manipulated by the media, thereby preventing themselves from getting caught in a negative emotional spiral [119]. Additionally, users should be mindful of the potential harm their personal statements might cause to others’ emotions, enhance their media literacy to prevent negative emotional contagion, and mitigate the potential adverse consequences.

#### Social media content management and interventions for public mental health

Relevant stakeholders could consider adopting the marketing approach used for commercial advertisements. They could periodically deliver "educational" or "guidance-oriented" public service information to users, aiming to promote and encourage users to adopt a proper perspective on and use of social media. Ultimately, through the responsible usage, development, and effective management of social media by individuals, businesses, and governments, a rationalization of online emotions, the promotion of healthy social media platforms, and the fostering of positive societal development can be achieved.

### Limitations and future research directions

This study aimed to focus on woman users who are among the most frequent and easily influenced by the context of social media usage. By combining in-depth interviews to gather authentic user experience data, we aimed to explore the dynamics between user emotions and media usage behaviors. While this approach contributes to enhancing the efficiency of social media feature development, management, and interventions for public mental health, there are still certain limitations to this research.

Firstly, although this study employed certain measures in the sampling process to ensure the diversity of the sample and the reliability and validity of subsequent analysis results, the final selection of twenty participants represents only a small portion of woman social media users in China. Moreover, these participants are concentrated in more developed regions and among the younger demographic with high social media usage and technological familiarity. Thus, the explanatory power of the research findings for rural areas, individuals with lower social media usage frequency, and older age groups needs further validation.

Secondly, this study employed in-depth interviews to explore the dynamic relationship between social media usage and emotions among Chinese women. However, the interview subjects were exclusively woman users from China, which limits the ability to compare media usage behaviors among different cultural groups and to analyze the impact of demographic variables such as age, education, and income on emotion perception and behavior. The study was unable to fully establish causal effects between emotions, media usage, or media dependence. Therefore, future research could consider combining qualitative and quantitative methods to comprehensively and objectively compare user social media usage behaviors across different cultural backgrounds and demographic variables. Engaging in a dialogue with the results of this study could further validate the generalizability and cultural differences. Exploring how media choice and usage frequency influence user emotions or considering emotions as mediators or moderating variables might shed light on their impact on users’ willingness for sustained usage.

Lastly, while in-depth interviews effectively avoid misjudgment of social media users’ genuine experiences that data mining might bring, they to some extent limit the generalizability of research findings. Future research could attempt to combine data mining of social media user data with in-depth interviews, examining the discrepancies and investigating their underlying causes. This approach could enhance the overall generalizability and authenticity of research results.

## Conclusions

This study aimed to explore the dynamic relationships among user emotions, satisfaction, and behavior based on the context of social media usage. It extended traditional attitude and behavior models by incorporating the social media context and the dimension of women’s emotions. Through in-depth interviews with woman social media users, personal experiential data were collected and used to construct a conceptual model, namely the Women’s Emotion-Satisfaction-Behavior Model in the context of social media usage. During the development phase of the conceptual model, analysis of user motivations, emotional states, satisfaction with social media usage, purposes, and behavioral manifestations was conducted based on the interview data. Subsequently, through axial coding and selective coding, thirty minor categories and three core categories were identified and deduced. Among these, emotional expression emerged as a primary motivation and purpose driving users to sustain their social media usage. Emotional masking represented a proactive behavior prompted by users’ need to maintain social relationships and accumulate social capital. Emotional management, on the other hand, referred to the act of users abandoning or shifting their social media engagement due to perceived emotional stress. Consequently, this study employed an experiential perspective of social media users, exploring the interplay of women’s emotions, satisfaction, and behavior. It aimed to investigate social media functionality enhancement, development, content management, as well as interventions for promoting public mental health, all within the context of the relationships highlighted above.
