# Correction: Emotional straying: Flux and management of women’s emotions in social media

**DOI:** 10.1371/journal.pone.0316285

**Published:** 2024-12-19

**Authors:** Pengpeng Li, Qianru Zhuo

The second affiliation of the first author should have not been indicated. Pengpeng Li is only affiliated with #1: Department of Shiliangcai Journalism and Communication School, Zhejiang Sci-Tech University; Hangzhou, Zhejiang, China.
